# Viral elements and their potential influence on microbial processes along the permanently stratified Cariaco Basin redoxcline

**DOI:** 10.1038/s41396-020-00739-3

**Published:** 2020-08-14

**Authors:** Paraskevi Mara, Dean Vik, Maria G. Pachiadaki, Elizabeth A. Suter, Bonnie Poulos, Gordon T. Taylor, Matthew B. Sullivan, Virginia P. Edgcomb

**Affiliations:** 1 Geology and Geophysics Department, Woods Hole Oceanographic Institution, Woods Hole, MA 02543 USA; 2 Department of Microbiology, The Ohio State University, Columbus, OH 43210 USA; 3 Biology Department, Woods Hole Oceanographic Institution, Woods Hole, MA 02543 USA; 4 Biology, Chemistry, and Environmental Studies Department, Molloy College, Rockville Centre, NY 11571 USA; 5 Department of Ecology and Evolutionary Biology, University of Arizona, Tucson, AZ 85716 USA; 6 School of Marine & Atmospheric Sciences, Stony Brook University, Stony Brook, NY 11794 USA; 7 Department of Civil, Environmental and Geodetic Engineering, The Ohio State University, Columbus, OH 43210 USA

**Keywords:** Microbial ecology, Metagenomics

## Abstract

Little is known about viruses in oxygen-deficient water columns (ODWCs). In surface ocean waters, viruses are known to act as gene vectors among susceptible hosts. Some of these genes may have metabolic functions and are thus termed auxiliary metabolic genes (AMGs). AMGs introduced to new hosts by viruses can enhance viral replication and/or potentially affect biogeochemical cycles by modulating key microbial pathways. Here we identify 748 viral populations that cluster into 94 genera along a vertical geochemical gradient in the Cariaco Basin, a permanently stratified and euxinic ocean basin. The viral communities in this ODWC appear to be relatively novel as 80 of these viral genera contained no reference viral sequences, likely due to the isolation and unique features of this system. We identify viral elements that encode AMGs implicated in distinctive processes, such as sulfur cycling, acetate fermentation, signal transduction, [Fe–S] formation, and N-glycosylation. These AMG-encoding viruses include two putative Mu-like viruses, and viral-like regions that may constitute degraded prophages that have been modified by transposable elements. Our results provide an insight into the ecological and biogeochemical impact of viruses oxygen-depleted and euxinic habitats.

## Introduction

Viruses are known to play key roles in the biogeochemistry of the global ocean by influencing nutrient cycling, respiration, particle sinking rates, biodiversity, and transfer of genetic information [1, 2]. Bacterial mortality due to viral infection in marine environments varies spatiotemporally and estimates lie between 10 and 50% of total mortality [2]. Viral infections can exert controls on species composition and activities of microorganisms [3] and can indirectly influence microbial metabolic fluxes, energy homeostasis, and metabolic reprogramming of the host cells [4]. For example, cyanoviruses have auxiliary metabolic genes (AMGs) that encode for core photosynthetic reaction centers [5] and these genes are expressed during infection to boost photosynthesis and increase viral abundance [6]. Virus-encoded AMGs are known to include genes involved with nearly all of central carbon metabolism [7], nitrogen [8], phosphorus [9] and sulfur cycling [10, 11], nucleotide metabolism [12–14], oxidative stress responses [15] and methane oxidation [16]. Even degraded prophages can reprogram metabolisms through altered gene regulation at the phage integration site [17] or by horizontal gene transfer enabling niche expansion among susceptible hosts [1]. Due to high energy costs, selection pressures, and physiological constraints, it is presumed that only maintain the most beneficial AMGs would persist in viral populations [18].

We know little about the ecology of viruses below the epipelagic zone, particularly in oxygen-deficient water columns (ODWCs). However, a few biochemically relevant AMGs have been identified in ODWCs, including an archaeal virus-encoded ammonia monooxygenase (*amoC*) and a SUP05 phage-encoded dissimilatory sulfite reductase subunit C gene (*dsrC*), among others [8, 19]. Redoxclines, or transitional zones between oxygenated and anoxic waters, provide a continuum of biologically important electron donors and acceptors, creating a diverse microbial niche space [20, 21], often harboring unique and low diversity viral communities with numerous endemic members [19, 22]. ODWCs are expanding and intensifying worldwide [23], and thus it is critical to understand how these changes shape microbial and viral populations and their activities.

The Cariaco Basin on the Venezuelan continental margin exhibits physically and chemically stratified waters below the mixed layer (<80 m) [24, 25]. The redoxcline extends from ~200 m down to ~250–350 m depth; below which the water becomes euxinic, with sulfide concentrations approaching 80 µM near the basin floor [26, 27]. Its bottom waters have remained anoxic and sulfidic for the past ~12,600 years [28]. Biogeochemical evidence suggests the deep euxinic zone harbors a predominantly heterotrophic microbial community, potentially involved in nitrogen and sulfur metabolism, and likely supported by fermentation, sulfur reduction, and methane metabolism [29–31]. Here, we explore the diversity of viruses detected in Cariaco Basin, as well as the variety of genetic elements detected within viral metagenomes prepared from water samples collected through the water column (ranging from fully oxygenated to euxinic) that may play roles in shaping prokaryotic metabolic activities.

## Materials and methods

### Water sampling

**Fig. 1:**
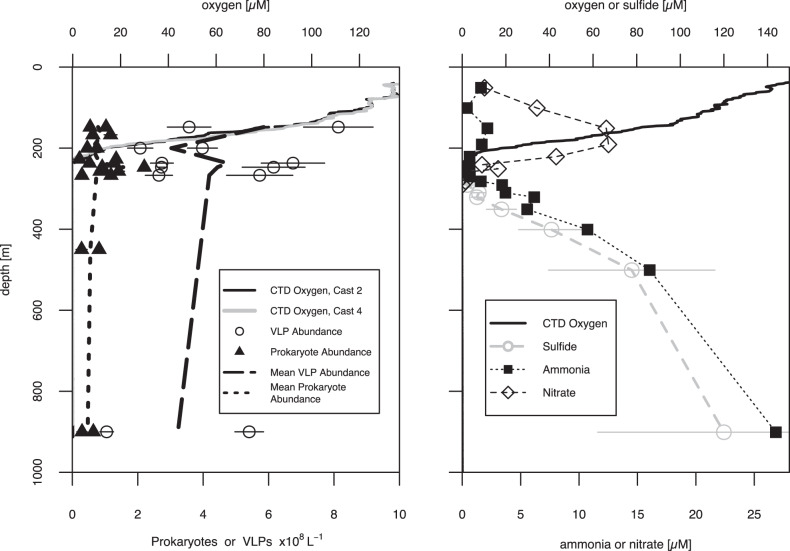
Biogeochemical data for the cruise when virome samples were collected (CAR216_2, left panel, 6–7 November 2014) and additional data collected 3 days later during cruise CAR216_3 (right panel, 10–11 November 2014). Virome samples were collected from casts 2 to 4 during CAR216_2 (left panel). Corresponding oxygen concentrations for the two casts from CTD sensors are presented as black lines and gray lines, respectively. Abundances of VLPs and prokaryotes from individual samples as measured by microscopy are presented as unfilled circles and filled triangles, respectively. Error bars for VLPs and prokaryotes represent the standard errors derived from counting multiple grids on the same filter. Average VLP (long-dashed line) and prokaryote (dotted line) abundances were calculated for duplicate samples. Additional samples were collected 3 days later during CAR216_3 for sulfide (gray dots), ammonia (black squares), and nitrate (black circles) (right panel). During CAR216_3 CTD oxygen profiles were similar (black line). Error bars for sulfide represent standard error among analytical triplicates from single preserved samples.

Hydrographic data and seawater samples from six depths at the Cariaco Basin Ocean Time-Series station (10.51°N, 64.67°W) were collected during CAR216_2 (6–7 November 2014) aboard the R/V *Hermano Gínes*. Hydrographic data for samples discussed are presented in Fig. 1 and Supplementary Table 1.

Depths sampled coincide with those targeted for previous studies of microbial communities (e.g., [32]). Sample collection, processing, and data treatment for O_2_, H_2_S, nutrients, microbial activity measurements and microscopic counts of prokaryotes and virus-like particles (VLPs) were performed as described in [33, 34]. Details on water sampling are provided as Supplementary Methods.

For the collection of VLPs, cells and particles were first removed by pre-filtering 10–18 L of seawater through a 0.22 μm Sterivex filter, retaining only the filtrate. Viral particles were then concentrated by FeCl_3_ flocculation, removed from suspension by filtration on a 142-mm diameter 1.0-μm polycarbonate membrane, and stored at 4 °C until further processing [35] (see Supplementary information for details).

### Virome processing and assembly

All bioinformatic analysis were conducted using the Ohio Supercomputer Center [36]. Viral particle metagenomes were prepared from oxic, redoxcline, and euxinic samples (Supplementary Table 1) according to methods in [37]. See full details in Supplementary Methods. The viral sequencing data were deposited in Sequence Read Archive (SRA), accession numbers: 148 m PRJNA375242 and PRJNA375241, 200 m PRJNA365439, 237 m PRJNA375245, 247 m PRJNA375239, 267 m PRJNA405926, 900 m PRJNA375240. Reads were quality trimmed with Trimmomatic v.0.33 to remove the Nextera adapters, low quality leading and trailing sequences, regions with a Phred score lower than 20 in a sliding window of 4 bp, and reads shorter than 50 bp [38]. Sequences from the two filters prepared from 148 m were co-assembled. Quality controlled reads for individual samples were assembled using Spades 3.11.1 with default settings and k-mer lengths of 21, 33, 55, and 77 nucleotides [39]. Contigs relevant to the data presented in this paper are deposited on Xenodo (temporary 10.5281/zenodo.3801713).

Reference and environmental viruses were selected for genome comparison with the AMG-encoding viruses by identifying those that fell into the same VconTact viral genera or those with the lowest *e*-value and/or highest number of BLASTp alignments using the RefSeq virus database with an *e*-value threshold of <0.0001. Genome alignments were then conducted by first creating GenBank files for each virus using Prokka v1.13 with the “–kingdom Viruses” option, which implements Prodigal for ORF prediction. Coding sequences (CDS) were then aligned between each virus using BLASTp as implemented by the Easyfig software version 2.2.2 [40] with a BLASTp *e*-value threshold of 0.0001. To determine whether AMGs on the flanking edges of a viral contig were part of the phage genome, DNA termini were predicted using PhageTerm and default settings [41]. AttL, attR, and putative prophage regions were predicted using PHASTER to provide additional lines of evidence to support the identification of viral genome boundaries. The read QC and assembly practices implemented in this study have been recently benchmarked by Roux et al. [42] who showed that assemblies >500 bp created by MetaSPADES, using quality-filtered reads, would result in a less than 2% chimeric or mis-assembly rate. This rate of assembly error is even lower for contigs with assembly coverage values higher than 5× which is the threshold applied to our data.

### Microbial metagenomes

Microbial metagenomes were prepared as described in [32]. See full details in Supplementary Methods. Microbial metagenome data were deposited in SRA, accession number PRJNA326482.

### Viral identification and annotation of viral and microbial genes

To identify viral sequences and to separate those from possible contaminating microbial sequences, we used a combination of four tools; VirSorter, VirFinder, Contig Annotation Tool (CAT) and PHASTER [43–46]. Viruses were identified here as in [47] with slight modification as follows. High confidence viruses were defined here as those in VirSorter categories 1 or 2 and those with a VirFinder score greater than 0.9 [43, 44]. Medium confidence viruses were those that were only identified by VirSorter or VirFinder, were predicted to be prophages by VirSorter (categories 4–6) and validated by PHASTER, or were identified in VirSorter’s category 3 and VirFinder with a score between 0.7 and 0.9 and were further validated to be viral by CAT [43–46]. Viral populations were established by clustering the contigs larger than 5 kbp at 95% average nucleotide identity over 80% of the shortest sequence using *nucmer* from the MUMmer 3.23 package [48]. The longest sequence in each cluster was used as the representative sequence of the population. Viral ORFs were predicted using Prodigal version 2.6.3 with the -p meta options [49]. Functional annotations for both viral populations and microbial contigs were provided as in [50] (see also Supplementary Methods).

### Putative AMG validation

Conserved domains and active sites of AMGs were identified using the NCBI conserved domain search (https://www.ncbi.nlm.nih.gov/Structure/cdd/wrpsb.cgi), and an *e*-value threshold of 0.001 (Supplementary Table 2). Noncoding intergenic regions (IGRs) and promoters were predicted using a python script (https://github.com/SBRG/sbaas/blob/master/sbaas/resources/get_interregions.py, [51] and the BPROM software [52, 53] (Supplementary Table 2). Descriptions of protein domains as well as protein structural homology of all viral elements were identified using the PROSITE database [54] and Phyre2 [55], respectively (Supplementary Table 2; Supplementary Methods).

Known viruses were identified by blastp against the RefSeq virus database using an *e*-value threshold of <0.0001. The reference virus represented by either the lowest *e*-value or highest number of alignments was selected for comparison with the AMG-encoding viruses. To determine whether AMGs on the flanking edges of a viral contig were part of the phage genome, DNA termini were predicted using PhageTerm and default settings [41]. AttL and attR sites predicted by PHASTER along with the PHASTER predicted prophage regions were used as additional lines of evidence to support the identification of viral genome boundaries. Genbank files for each contig encoding a viral element were created using Prokka v1.13 with the –kingdom Viruses option. CDS were visualized using Easyfig version 2.2.2 [40].

### Ecological analyses of viral data

**Fig. 2:**
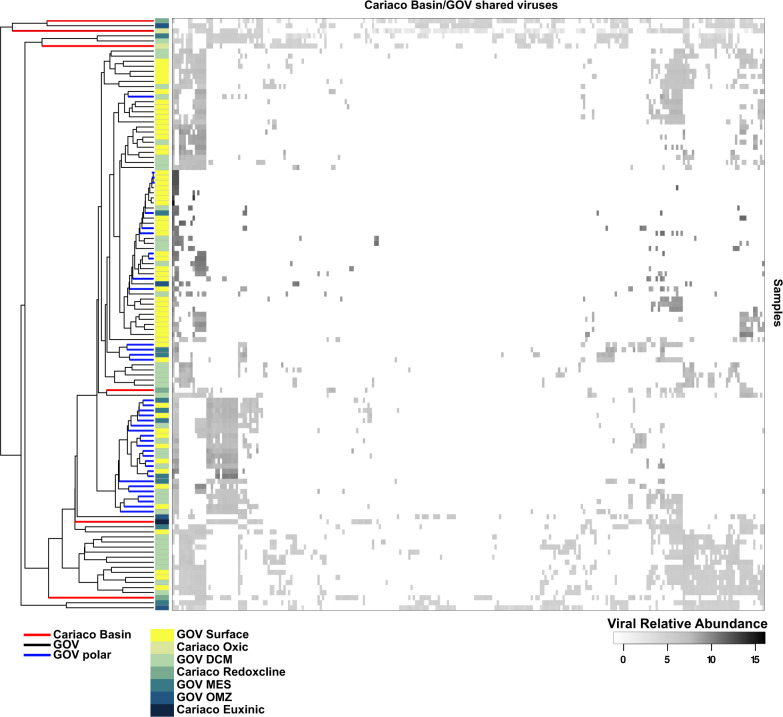
Hierarchical clustering of the normalized relative abundances of viral populations across each as identified in the Tara Oceans dataset. Each row represents an individual virome, labeled with the sample name, depth and oceanographic feature. Each column represents an individual viral population (≥5 kbp), where the normalized relative abundance values (ln transformed) are shown in grayscale. Samples from Cariaco Basin are labeled in red.

Viral populations present in the Cariaco Basin, but not recovered by the assemblies were identified by recruiting the Cariaco Basin paired and non-paired end reads to the 488 k viral populations larger than 5 kb identified in the Tara Oceans dataset [47]. Coverage values were only retained for contigs which recruited reads to over 75% of the contig at a read identity of 95% over 90% of the read. These coverage values were then normalized by metagenome size and contig length to derive a proxy for relative abundance, which in turn was used to evaluate the local and global distributions of the identified viral populations (Fig. 2). Expanded details are described in Supplementary Methods.

The degree of sample saturation for the Cariaco Basin samples was calculated in R version 3.4.4 with the R specaccum package using 100 permutations and the jackknife 2 richness estimator (Supplementary Fig. 1) [56]. Nonmetric multidimensional scaling ordinations of the samples used Bray–Curtis dissimilarities to discern relationships among samples based on viral relative abundances (Supplementary Fig. 2) [56]. Cariaco viromes were hierarchically clustered alone and then together with the second Global Oceans Virome from the Tara Oceans datasets (GOV2.0) using the R package pvclust [57] and Manhattan distances with 100 permutations (Fig. 2). The distribution of identified viruses was plotted using the R package heatmap3 (Fig. 2).

### Viral taxonomic assignments

Genus scale taxonomic assignments were applied to identified viral populations larger than 10kbp using VconTACT2 [58]. Viral ORFs along with a text file linking each ORF to a contig were uploaded to VconTACT2. NCBI RefSeq v.85 was used to classify specific viral genera. Specific connections and taxonomic affiliations are available in Supplementary Table 3.

## Results and discussion

Viral particle abundances tracked prokaryotic abundance through the Cariaco water column with greatest abundances at 148 m and within the redoxcline (~237–267 m depth, Fig. 1; Supplementary Table 1). From all six samples we sequenced a total of 1.5 M reads, 2 orders of magnitude more sequencing depth than previously derived from any other ODWC viromes [22] and ~70% of sample sequencing depth for recent surface ocean viromes [11, 47]. From these samples we identify 2232 high and medium confidence viral sequences larger than 1.5 kbp (See Supplementary information for details).

Taxonomic clustering of viral sequence space into species-level delineations, designated as populations, is well established by gene flow studies and population genetics theory [47, 59–61] (See Supplementary Information for expanded discussion). Viral populations are defined as viral sequences that cluster at 95% identity over 80% of the shorter sequence and are larger than 5 kbp [47]. From the Cariaco Basin viromes, 150 million quality trimmed reads were recovered which assembled into nearly 1 million contigs. Viral identification and population-scale clustering as defined above yielded only 2232 clustered viral sequences with only 647 larger than 5 kb, thus meeting the requirements to be termed populations representing distinct ecological units (Supplementary Fig. 3; Supplementary Table 4). Comparable community-based viral species counts from other ODWCs are not currently available. However, the number of populations we recovered is ~25% of the number of populations recovered from other viromes in the surface ocean [47]. By recruiting Cariaco reads to the Global Ocean Viromes (GOV) 2.0 dataset [47] we detected an additional 101 viral populations. In total, we recovered 748 viral populations, which recruited on average, 3% (range 0.7–6.5%) of the reads from the pooled Cariaco Basin viromes, with the remaining reads being not detectably viral, possibly representing cellular contamination or novel viruses that failed to assemble (Supplementary Tables 4 and 5). This is consistent with other ocean virome studies [7, 62], but lower than what was achieved by two generations of Tara Oceans virome analyses [11, 47]. The proportion of viral reads in each sample may reflect a viral community comprised of viruses unique to the Cariaco Basin and possibly to other ODWCs (Supplementary Fig. 3; Supplementary Table 4). Read recruitment to all phage sequences described below also reveals generally consistent coverage across all phages (unless otherwise noted), indicating no assembly error. Host predictions for the recovered viral populations were attempted using k-mer frequency comparisons, CRISPR spacer matches, and tRNA comparisons, however, no statistically robust results were obtained.

A gene-sharing network analysis identified 94 viral genera comprised of 313 viral populations with 116 outliers (assigned to a cluster but sharing relatively fewer proteins), and 319 singletons (Supplementary Table 3). Of the 94 clustered viral genera, 14 were associated with bacteriophages infecting *Cellulophaga, Acinetobacter*, and *Pseudomonas*, bacteria detected in 16S rRNA libraries from the same water samples [32] and 80 had no known reference viruses and represent novel viruses (Supplementary Table 3). The AMG-containing viral contigs likely represent novel viruses at a level greater than genus because of their lack of clustering in the gene-sharing network analysis, and we cannot evaluate them further using marker genes as these contigs lack such marker genes.

Of the 748 Cariaco populations, 219 were also found in the Tara Oceans dataset, and 529 appeared to be present only in Cariaco Basin indicating a relatively high degree of endemism among the identified viral populations (Supplementary Table 5). Of the viral sequences endemic to Cariaco, 217 were only detected in anoxic habitats with 177 of these being exclusively found in the euxinic zone, 28 only detected in the anoxic redoxcline, 11 found in both the redoxcline and euxinic zone, and 53 populations had near undetectable abundances, limiting inference on their distribution. Among the 219 populations shared with the Tara Oceans dataset, 122 were also shared only among the oxygenated habitats in the Cariaco Basin indicating a more cosmopolitan lifestyle for these populations. Only nine populations shared with the Tara Oceans dataset were found to be exclusive to the euxinic zone in Cariaco Basin. These nine populations were also found in 27 Tara Oceans stations, 26 of which were from “Tara Polar” which encompasses samples from within the Arctic circle and one from the ODWC in the Arabian Sea (Supplementary Table 5).

While hierarchical clustering is challenging with low sample saturation, observed clustering between our samples and those from the Tara Oceans GOV2.0 dataset are likely driven by nitrate and oxygen concentrations (samples from 200 to 237 m depth with Tara Oceans station 38_MES) and low species richness and alpha diversity (sample from 267 m with Tara Oceans station 32_DCM) [47]. Two of our samples (247 and 900 m) do not cluster with any other sample, likely reflecting novel communities, however low sampling saturation should be taken into consideration (Fig. 2).

Sample saturation analyses based upon accumulation curves imply the bulk of the viral community in each sample remains unidentified with a >38% new population detection rate in the final random subsampling (Supplementary Fig. 1). Relative population composition and abundance displayed a high degree of evenness (Pielou’s J 0.997–0.999) indicating a low proportion of dominant populations in each sample. The highest observed species richness and alpha diversity were found in the euxinic zone at 900 m, followed by the redoxcline samples from 237 m, oxic sample from 200 m, the redoxcline samples from 247 m, the oxic sample at 148 m, and finally the redoxcline sample from 267 m (Supplementary Table 4). These indices must be interpreted with caution because diversity estimates are heavily influenced by the degree of sample saturation and sequencing depth (Supplementary Figs. 1 and 3) [63]. Nonetheless, results are roughly similar to those for bacteria and archaea in Cariaco [32] where diversity was highest in oxic and euxinic samples and lowest in the redoxcline, suggesting viral diversity might be driven by the diversity of microbial hosts. Ordination analysis with Bray–Curtis dissimilarities revealed no statistically significant patterns among distributions of viral groups from different samples. The only exception was a very tight association between 237 and 247 m samples (Supplementary Fig. 2).

Composition of viral populations in the Cariaco Basin relative to the GOV 2.0 dataset appears to include groups that are similar to those found in other deep ocean regions around the world, but also groups in its anoxic and euxinic waters not detected previously. This is likely due in part to under-sampling of euxinic waters globally, so it would be premature to draw conclusions about the novelty of Cariaco’s viral community. We focus further attention on the genetic content of the viral populations we detected.

### Potential auxiliary metabolic genes (AMGs)

**Fig. 3:**
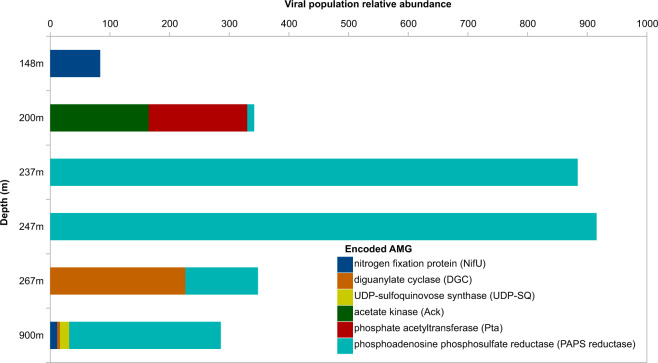
Viral population relative abundance of AMGs along the water column in the Cariaco Basin. Relative abundance of AMG-encoding viral populations (coverage values normalized by metagenome size and contig length) detected in viromes from different depths along the water column in the Cariaco Basin.

Marine viruses were found to encode metabolic genes of host origin which may be retained in the viral genome if they enhance production of new viruses by bolstering metabolism of their hosts [1, 7, 64]. Viral metagenomes from the surface and upper oxycline of the Eastern Tropical South Pacific (ETSP) ODWC contained bacterial genes involved in many metabolic processes [22]. Metabolic genes in viral communities may alleviate efficiency bottlenecks in the metabolisms of infected hosts. Evidence for this comes from viruses mined from SUP05 genomes from the Saanich inlet coastal ODWC which encode bacterial genes involved in phosphate, nitrogen, and sulfur metabolism [19]. Viruses identified in the Cariaco Basin carried genes implicated in biochemical pathways that were expected to be active at several or all depths (Fig. 3, Supplementary Table 2). By examining gene content and organization within the Cariaco viromes, we predict whether these elements are probable AMGs.

### Assimilatory phosphoadenosine 5′ phosphosulfate (PAPS) reductase

**Fig. 4:**
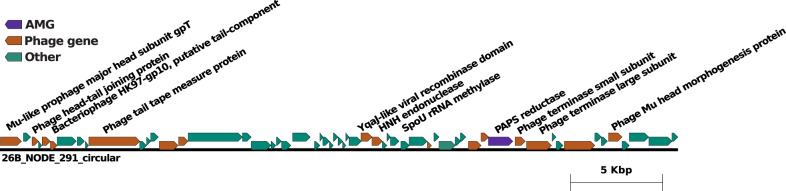
Genome map of the putative PAPS AMGs. Genome map of the two representative PAPS reductase encoding viruses, displaying the AMG of interest in purple, genes observed in other viromes as indicated by VirSorter in orange, and non-phage like or uncharacterized genes in teal.

PAPS reductase is an enzyme in sulfur metabolism and was detected in almost all our viromes except the oxic sample at 148 m, with the greatest diversity of PAPS reductase domains detected at 900m. We identified nine viral contigs encoding PAPS reductase genes (Supplementary Table 2), three of which cluster into distinct viral genera with four other viral populations, and six that are classified as singletons or outliers in our gene-sharing network analysis. Each of the PAPS reductases encoding contigs contained clear viral genes indicating a true viral origin for the PAPS gene. The best representative of these viruses is shown in Fig. 4. Both VirSorter and PHASTER place the PAPS reductase encoding genes within the interior of the viral genome. However, no attL/R sites or termini regions were identifiable, indicating incomplete viral genome recovery.

All representative PAPS reductase genes bear the expected conserved domain and structural configuration of PAPS reductase (Supplementary Table 2) that assimilates sulfates for two essential amino acids (methionine and cysteine) in both aerobic and anaerobic organisms [65]. See Supplementary Information for discussion of PAPS conserved domain and structural homologies.

In microbial metagenomes from water samples collected concurrently with virome samples, we found PAPS reductase genes. However, only one gene from *Clostridiales* was closely related to a viral PAPS. This suggests multiple origins and/or evolutionary histories of the viral PAPS reductase sequences in the Cariaco Basin.

The PAPS reductase detected in our Cariaco viromes is the first detected putative AMG directly involved in assimilatory sulfur metabolism linked to amino acid biosynthesis. A study of *Sulfurimonas* concluded that PAPS reductase provides metabolic scope to adapt to variable redox conditions [66]. We hypothesize that PAPS reductase can enhance biosynthesis of methionine and cysteine for protein synthesis. Additionally, in the Cariaco Basin’s euxinic interior, where sources of labile carbon are limited, bacteria can benefit by fermenting amino acids produced from intermediate products (e.g., pyruvate) of methionine and cysteine degradation. Thus, we hypothesize that PAPS reductase enhances the metabolic flexibility of this sulfur-driven microbial food web.

### AMGs from Mu-like phages

Mu-like phages represent an intriguing example of viruses that can persist through replicative transposition within the host genome [67–69]. Multiple Mu-like phages have previously been resolved that include 0.5–3 kb of host DNA covalently bound to the edges of their genome during headful packaging [70–72]. Thus, Mu-like viruses can acquire and mobilize host genes among susceptible hosts [73, 74]. Distinguishing host gene acquisition from randomly packaged host genomic material carried by Mu-like viruses is challenging. Typically, randomly packaged host genes will be discarded, and are not likely to be detected by population-scale metagenomic screens. However, genes may be maintained in the viral population if they provide a selective advantage [75]. Identification of host metabolic genes in the interior of a phage genome representing a population-scale cluster of viral contigs would provide evidence for the maintenance of such genes.

**Fig. 5:**
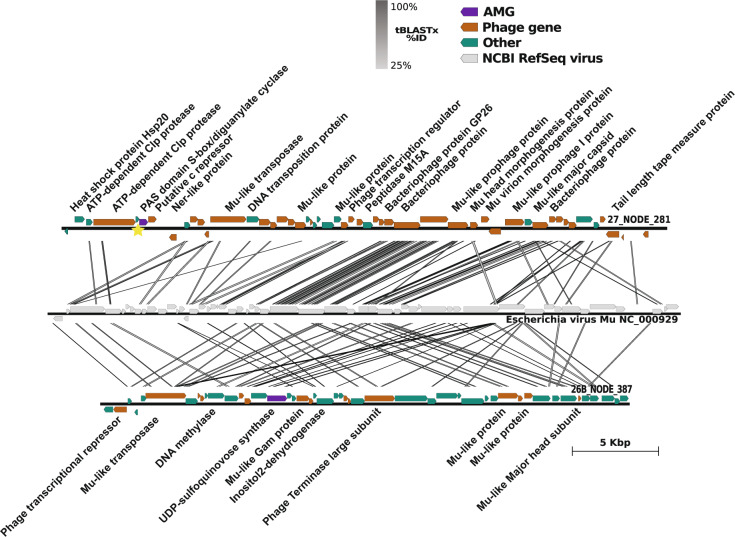
Genome maps of the probable  Mu-like phage AMGs. The representative DGC (upper genome map) and the representative UDP-SQ encoding contigs (lower genome map) display the gene of interest in purple. Genes observed in other viromes as indicated by VirSorter are in orange and the non-phage like or uncharacterized genes in teal. The yellow star at the DGC genomap shows a predicted promoter site. Both the DGC and UDP-SQ encoding viruses also encode Mu-like transposase. An alignment of these viruses with *Escherichia* virus Mu, reveals a high degree of similarity with DGC and UDP-SQ viruses.

We identified two probable  Mu-like phages encoding putative AMGs involved in signaling pathways and N-glycosylation (Fig. 5). Both share a high degree of syntenic arrangement with Bacteriophage Mu along with numerous short homologous regions (BLASTp *e*-value <0.0001). While each of these short homologous regions is not individually compelling, the number of these hits, the proportion of genes annotated as Mu-like, and the syntenic arrangement of these genes suggests that these may be novel Mu-like viruses. The first Mu-like virus, encoding diguanylate cyclase (DGC) involved in signaling pathways, was found in the sample from 267 m where it comprised ~2% of the total observed viral community and was 76% as abundant as the most abundant population (Fig. 3 and extended discussion in Supplementary Information). We detected a second Mu-like virus encoding a putative UDP-sulfoquinovose synthase, in euxinic waters at 900 m where it was less than 1% of the total community and 55% as abundant as the most abundant population (Fig. 3). Each of these viruses encode diagnostic Mu-like proteins, including Mu-like major capsid and morphogenesis proteins. Other genes, with non-viral homology include multiple uncharacterized proteins, an ATP dependent clp protease, and transposon B, with the last two being cellular genes that have been shown to play a role in Mu-like virus activation [76]. One of these Mu-like viruses, encodes the cellular Clp protease, DCG, and the phage c repressor at the edge of the viral contig, drawing into question whether the DCG is part of the phage or host genome. However, this region, spanning both cellular and phage genes, had consistent coverage (albeit higher than the rest of the sequence) which in combination with the lack of an identifiable att site and the presence of a promoter upstream of the DCG, are indicative of a contiguous region without a phage genome boundary. The higher coverage of this region is likely due to our population-scale clustering, allowing reads from different subpopulations to accumulate on the representative contig. The high degree of similarity with bacteriophage Mu and the presence of Mu-like transposases, along with other proteins important for Mu activation, suggest that these phages are indeed Mu-like rather than degraded prophage regions encoding a non-phage transposable element. A third Mu-like virus was identified in the 900 m sample, but did not encode any detectable AMGs (see Supplementary Information).

#### Diguanylate cyclase

DGCs have been detected in viromes from the Pacific Ocean and linked to signal transduction mechanisms [77]. Genes associated with cell signaling were also detected in viromes from the surface and oxycline waters of the ETSP ODWC [22], and in cultivated viral isolates [78]. Selective pressure may exist to retain DGC genes in viral elements since they can enhance rates of conjugative plasmid transfer in anaerobic bacterial strains via the production of the secondary messenger cyclic diguanylate (c-di-GMP) [79]. This could enhance host fitness in ODWCs by increasing gene transfer. c-di-GMP is a signaling molecule that also induces biofilm formation [80]. Since particle-associated microbes play an important role in the Cariaco water column [32, 81], viral-encoded DGCs may enhance signal transduction involved in biofilm formation. See Supplementary Information for discussion of viral-encoded DCG function and structural homology.

#### UDP-sulfoquinovose synthase

Viral elements related to glycosylation pathways may contribute to viral fitness by increasing host protein stability or by increasing production of intermediate substrates (e.g., oligosaccharides) [82] that can be utilized by hosts in the euxinic interior of Cariaco Basin. Elevated hydrostatic pressure, much like temperature, can cause protein transitions between native and unfolded states [83]. N-glycosylation was found to decrease dynamic fluctuation of proteins and to increase stability [84]. See Supplementary Information for discussion of viral-encoded UDP-SQ function and structural homology.

Genes related to N-linked glycosylation are encoded in almost all archaeal genomes obtained to date, and in a small number of bacterial species [85]. N-glycosylation is a common posttranslational modification that promotes and regulates protein folding [86] and is considered essential for maintaining cell integrity under extremes of temperature, pH, salinity as well as other physical challenges [87]. N-glycosylation in bacteria, is related to protein thermostability in extreme environments, such as hydrothermal vents [85]. We hypothesize that viral-induced changes in host N-glycosylation pathways via this viral-borne UDP-SQ AMG may improve viral fitness by enhancing host protein stability under the pressures encountered at 900 m in the Cariaco Basin.

### Other phage-related viral elements detected in the cariaco basin

#### Acetate metabolism

**Fig. 6:**
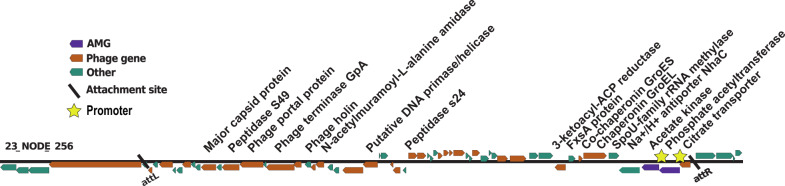
Genome map of the representative Ack and Pta encoding contig, displaying the AMG of interest in purple, genes observed in other viromes as indicated by VirSorter in orange, and non-phage like or uncharacterized genes in teal. The yellow stars show predicted promoter sites. PHASTER identified both the attL and attR attachment sites indicated by the black bars and denoting probable phage genome boundaries.

Viral elements related to acetate metabolism were detected from Cariaco’s oxycline at 200 m. One viral contig, predicted by PHASTER to be a complete prophage bounded by both attL and attR sites, encoded both an adenine phosphoribosyltransferase (Pta) and an acetate kinase (Ack) (Fig. 6). The Ack/Pta pathway mediates acetate fermentation [88] and is the major regulator of the acetyl-phosphate levels which control protein acetylation in bacteria [89]. Both genes were adjacent to each other on the contig. The contig also encodes 28 additional phage-like genes including multiple hallmark genes and aligned with Vibrio phage ×29 (NC_024369), suggesting a viral origin (Fig. 6). This phage may have picked up cellular genes on one end of this contig, such as the chaperonins GroES and EL. However, both phages and archaeal viruses are also known to encode chaperonins to assist in structural protein folding during infection when the expression of these genes is high [90–93].

The potential importance of acetate as a carbon source in the Cariaco Basin is intriguing, as acetate cycling has been shown to vary seasonally and vertically [94]. Observed acetate uptake rate constants were highest in the euphotic zone and at the suboxic–anoxic boundary (0.03–1.4 d^−1^) and diminished below 400 m (<0.01 d^−1^) [94]. Paradoxically, the *Pta* and *Ack* genes on viral contigs were only detected in our viromes from 200 m. Acetate is typically released to the environment by fermentative bacteria which are not expected to be active where oxygen is still present. However, enriched acetate concentrations have been observed in Cariaco oxic waters between 200 and 300 m on multiple occasions [94]. Particle-associated anoxic microenvironments in this layer may be conducive to fermentation and acetate release [32]. We hypothesize *Ack* and *Pta* genes on viral contigs may influence host metabolism in Cariaco waters by altering host metabolic flux and energy homeostasis or by increasing the pool of available acetyl phosphate and thus rates of acetyl phosphate-dependent acetylation. Both may lead to increased viral fitness by providing additional ATP and can support use of alternative carbon sources [7].

#### Iron–sulfur cluster formation

AMGs related to the iron–sulfur cluster and sulfur mobilization [Fe–S] formation systems have been previously described in viromes from the Pacific Ocean’s photic zone [77] and from the Global Ocean Survey data sets [95], suggesting that supporting host electron transfer enhances viral replication success. In the nitrogen fixation (NIF) system, NifU and NifS work in concert to synthesize the oxygen-sensitive [Fe–S] clusters required for the activation of nitrogenase [96] and are also involved in the biosynthesis of the iron–molybdenum (Fe–Mo) cofactor [97].

**Fig. 7:**
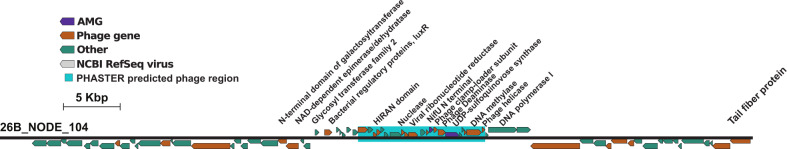
Genome map of the representative NifU encoding contig, which also encodes another UDP-SQ gene. The AMG of interest is displayed in purple, genes observed in other viromes as indicated by VirSorter are in orange, and non-phage like or uncharacterized genes in teal. No attachment sites delineating genome boundaries were identified, however PHASTER did identify a specific phage like region (light-teal).

Two viral contigs found in samples from 148 and 900 m encoded a putative NifU gene (Supplementary Table 2). The shorter of these contigs only encoded six genes. Two of these genes were similar to those in *Pelagibacter* phages, but there was little other support for a viral origin of this population. On the larger of these contigs, the *nifU* gene was located in the center of the contig and was surrounded by both phage-like and bacterial genes (Fig. 7). This NifU gene also contains an NifU conserved domain and shares secondary structural homology with described NifU-C domains (91.3% confidence, 30%ID) (Supplementary Table 2). Of the 74 predicted proteins, 18 were observed in other viruses. This contig encodes at least one viral tail fiber protein and a phage-like HIRAN domain (Fig. 7). HIRAN domains are DNA-binding domains that recognize DNA damage and stalled replication forks [98]. Although they have been identified in phages, their function in phages remains unclear [99]. It is likely this represents a region within a larger phage genome that may influence lipopolysaccharide biosynthesis. These phages often contain sugar epimerase, transferase, and synthase genes [12]. However, it is also possible that this putative AMG is in a cellular region bordering a prophage in a cellular genome. Regarding the latter, PHASTER identified the specific NifU encoding region as a putative prophage, supporting the likelihood that this is indeed a phage-encoded NifU gene. However, no attL, attR, or termini regions were identifiable (Fig. 7).

### The “mobilome”

Degraded prophage regions are often hotspots for mobile element activity [100] and so may represent biogeochemically relevant phage-like regions that carry metabolically active genes derived from transposable elements and not the viral genome. In the present study, a putative transposable element encoding a cysteine desulfurase (*nifS*) gene was found in viromes from nearly all depths sampled (Supplementary Table 2). NifS may be important in low redox environments because it can enhance electron transport and influence the activity of proteins that boost metabolism and fitness of the host. Abundance of *nifS* was especially high in the oxic sample, indicating a possible cyanobacterial association. The *nifS* gene in this putative mobile element includes the *nifS* conserved domain (Supplementary Table 2) and is flanked on one side by a phage-like integrase and numerous phage-like transposase genes (Supplementary Fig. 4) thus presenting the possibility of a viral origin. However, due to small size of this sequence and the multiple transposon genes (transposase mutator and transposase) this sequence may also be a phage-like transposable element in a degraded prophage region of the cellular genome. Thus, it is not possible to confidently link this *nifS* gene to the remnant prophage or the transposon.

## Conclusions

Viromes recovered from Cariaco Basin water samples reveal viral communities composed of a high proportion of unique viruses. We detected viral elements potentially contributing to a wide range of metabolic processes in their hosts. Some of these genes support central metabolism, and others support processes that occur in putative host populations in geochemical regimes specific to oxygen-depleted habitats. While some elements can be acquired by viruses through random packaging of host genetic material prior to host lysis, we report the presence of bacterial genes that would enhance or stimulate particular host metabolic processes, resulting in increased production of raw materials required for formation of new viral particles.

## Supplementary information

Supplementary Information

Supplementary Figure 1

Supplementary Figure 2

Supplementary Figure 3

Supplementary Figure 4

Supplementary Table 1

Supplementary Table 2

Supplementary Table 3

Supplementary Table 4

Supplementary Table 5
