# Supplementary material for: Viral elements and their potential influence on microbial processes along the permanently stratified Cariaco Basin redoxcline
Source: ISME J. 2020 Aug 14;14(12):3079–92. doi: 10.1038/s41396-020-00739-3 (PMC7785012; doi:10.1038/s41396-020-00739-3)
Supplement: Supplementary file 1 — Supplementary Information [file 41396_2020_739_MOESM1_ESM.docx]

Supplementary Information

Viral elements and their potential influence on microbial processes along the permanently-stratified Cariaco Basin redoxcline.

Mara, P.^†1^, Vik, D.^†2^, Pachiadaki, M.G.^3^, Suter, E.A.^4^, Poulos, B.^5^, Taylor, G.T.^6^, Sullivan, M.B.^2,7^, Edgcomb V.P.^1^

^1^Geology and Geophysics Department, Woods Hole Oceanographic Institution, Woods Hole, MA 02543

^2^Department of Microbiology, The Ohio State University, Columbus, OH 43210, USA

^3^Biology Department, Woods Hole Oceanographic Institution, Woods Hole, MA 02543, USA

^4^Biology, Chemistry, and Environmental Studies Department, Molloy College, Rockville Centre, NY 11571, USA

^5^Department of Ecology and Evolutionary Biology, University of Arizona, Tucson, AZ 85716, USA

^6^School of Marine & Atmospheric Sciences, Stony Brook University, Stony Brook, NY 11794, USA

^7^Department of Civil, Environmental and Geodetic Engineering, The Ohio State University, Columbus, OH 43210, USA

†Co-first authors

Supplementary Figure 1. Accumulation curve accounting for 100 random permutations of the viral populations identified in the 6 viromes and indicating a non-saturated sampling effort as represented by the non-asymptotic shape of the curve. Richness was estimated using the Jacknife2 algorithm.

Supplementary Figure 2. Non-metric multidimensional scaling analysis of the viral populations using Bray-Curtis dissimilarities. Samples are color coded according to their origin in either the oxic region in blue, redoxcline region in green or euxinic zone in red.

Supplementary Figure 3. Sequencing depth in number of total paired reads per virome. The grey bars represent the number of paired-end reads used for read recruitment while the black bar represents the number of paired end reads that recruited the pooled viral populations.

Supplementary Figure 4. “Mobilome” metabolic genes on a phage like transposase. Genome map of the representative cysteine desulfurase NifS encoding contig, displaying the gene of interest in purple, genes observed in other viromes as indicated by VirSorter in orange, and non-phage like or uncharacterized genes in teal.

Supplementary Table 1. Biogeochemical settings for the six viral samples collected along the water column in the Cariaco Basin.

Supplementary Table 2. Relative abundances, conserved domains, protein structures and predicted number of promoters for the AMGs detected at different depths from the oxic, redoxcline and euxinic interior in the Cariaco Basin.

Supplementary Table 3. Order, family, and genus scale taxonomic association and confidence thresholds for the taxonomic association, for each viral population as indicated by VconTACTv2.

Supplementary Table 4. Ecology summary values for the oxic (148, 200m), euxinic (900 m) and redoxcline (237, 247, 267m) samples.

Supplementary Table 5. Natural log scaled, sequence length, and metagenome size normalized viral population relative abundances. Populations with low to undetectable abundances have been removed by our read recruitment thresholds (see methods). Populations and samples derived from the GOV2 dataset are labeled with “GOV2”.

**Supplementary Material and Methods**

***Water Sampling***

Water samples were collected in Teflon-coated 8 or 12L Niskin bottles. Temperature, salinity, dissolved oxygen, and turbidity as transmissometer beam attenuation (BAT) were profiled with a Seabird SBE 25 conductivity-temperature-depth (CTD), an SBE 43 oxygen probe, and C-star transmissometer (WetLabs) mounted on a General Oceanics rosette.

At each depth, whole water samples were preserved with 2% glutaraldehyde (final conc.) and frozen immediately after collection in the field. In the laboratory, 1-3ml of preserved samples were filtered onto 0.02µm Anodisk membranes (Whatman), stained with SYBR Gold (Molecular Probes Inc.), and VLPs were enumerated by epifluorescence microscopy at 1000x magnification according to [1].

CTD data were collected and processed according to methods used by the CARIACO Ocean Time-Series program [2]. Biogeochemical data were collected from the same depths during the CAR216_3 time-series cruise several days later (10-11 November 2014) and are available at <http://www.bco-dmo.org/project/562425>[.](file:///C:\home\dean\Desktop\Supplementary%20Information_Virus%20paper_DV.docx)

***Viral particle collection***

Three of the six samples were further purified by cesium chloride (CsCl) gradient ultracentrifugation due to the presence of excessively high concentrations of DNA indicating cellular contamination (Supplementary Table 1). Cellular contamination in viromes for AMG studies requires caution to avoid presence of cellular sequences in the viral contigs [3,4]. However, we previously evaluated the impact of different purification methods (0.2um filtration + DNAse, 0.2um filtration + CsCl purification + DNAse, 0.2um filtration + sucrose purification + DNAse) and showed these via replicated metagenomes to be taxonomically indistinguishable where done well [5].

Viral particles from all collected samples were resuspended in ascorbic-EDTA buffer (0.1 M EDTA, 0.2M MgCl_2_, 0.2 M ascorbic acid, pH 6.0), concentrated using Amicon Ultra 100kDa centrifugal filter columns, and treated with DNase I (100 U ml^-1^) [6]. Viral dsDNA was then extracted with a Wizard PCR prep DNA purification kit (Promega) using 1ml resin to 0.5ml sample, and eluted in Tris-EDTA (10mM TRIS, pH7.5, 1mM EDTA) with Wizard Minicolumns. DNA was then submitted to JGI for library prep using a Nextera Truseq kit and sequencing on 1/8th of a HiSeq 1T lane.

***Microbial metagenomes***

Niskin bottle-collected water samples were used for DNA analyses to construct prokaryote metagenomes. Samples were filtered sequentially through EMD Millipore 2.7 µm glass fiber filters, then through 0.2 µm Sterivex filters. DNA from both glass fiber and Sterivex filters was extracted according to [7,8] described in detail in [9]. After extraction, DNA was purified with the Genomic DNA Clean and Concentrator-25 kit (Zymo Research), eluted into 10mM Tris-HCl, and frozen until downstream analysis. An aliquot of the extracted DNA was sent to Georgia Genomics Facility for library preparation and sequencing. The Illumina NextSeq platform was used for paired-end 2x150 sequencing. The R1 and R2 reads were filtered using Trimmomatic [10], which performs a “sliding window” trimming, removing sequence data once the average quality within the window (8 nucleotides used here) falls below a threshold (set to 12). The length of the trimmed sequences was set to a minimum of 50 nucleotides. The trimmed reads were assembled into contigs using IDBA 1.1.1 [11].

***Annotation of viral and microbial genes***

Open reading frames (ORFs) were first predicted using Prodigal v2.6.2 with the “meta” option [12]. Sequences were then compared with KEGG, Uniref90, and InterProScan (IPS) using USEARCH to identify individual and reciprocal best hits (RBHs) above a bitscore threshold of 60 [13,14,15,16]. The annotation qualities were ranked on a scale from A to E with RBHs to the KEGG database given rank A, RBHs to Uniref90 given rank B, individual best hits to KEGG given rank C, individual best hits to Uniref90 given rank D, and annotations with hits only to IPS given rank E [17]. Predicted viral genes previously identified as auxiliary metabolic genes (AMG) [18] and genes involved with a microbial metabolic pathway were manually mined from the annotations using Pfam accessions. We report putative AMGs only for contigs that were larger than 5kbp, and that also include clearly-identified viral genes. Microbial metagenomes were functionally annotated in the same manner as the viromes. Genes identified as putative AMGs in the viromes were matched to genes in the microbial metagenomes.

***Putative AMG validation***

Promoters were predicted using the BPROM software. BPROM returns positions for all identified promoters in the input sequence utilizing a set of seven features that include five relatively conserved sequence motifs, represented by their weight matrices, the distance between −10 and −35 elements and the ratio of densities of octa-nucleotides overrepresented in known bacterial transcription factor binding sites relative to their occurrence in the coding regions [19]. For each identified promoter a linear discriminant function (LDF) score is provided as a measure of the promoter strength; a predicted promoter is considered active when the LDF score has a value of at least 0.2. [20,21]. Promoter prediction in the intergenic regions used BPROM with 80% accuracy.

Regarding the descriptions of protein domains, PROSITE hits were automatically scanned against the pattern’s associated “miniprofile” data that assign a status to pattern matches. Structural homologies of the predicted proteins from viral elements were analyzed using models generated by Phyre2 using a confidence threshold of 98% and an identity threshold of 15%. The accuracy of the models constructed using Phyre2 is described as extremely high when the sequence identity is above 30-40%. However, lower sequence identities can be accurate as long as the confidence is high, which was the case in all of our examined viral elements.

***Ecological analyses of viral data***

Tara Oceans viral populations were included in our dataset if at least 75% of the contig length had >5x coverage and if they were larger than 5kbp. Relative abundance and local distributions of the viral populations larger than 5 kb were estimated by recruiting, paired and non-paired end, quality trimmed reads from the Cariaco Basin viromes. Global distributions of the viral populations identified in Cariaco Basin were assessed by recruiting reads from all of the Tara Oceans viromes. All read recruitment was done using Bowtie2 version 2.3.4.1 and default settings [22]. The resulting coverage values for the relative abundance, local distributions, and global distributions of the viral populations were then calculated using a wrapper script for BamM parse using the trimmed pileup coverages, normalized to the length of the viral contig and the number of reads recruited per virome, and transformed by ln(x+1) (https://github.com/ecogenomics/BamM) (Supplementary Figure 1).

**Supplementary Results and Discussion**

The term “viral population” has been well established by gene flow studies and population genetics theory. More recently, permissive read recruitment using a large and diverse viral dataset (Tara Oceans Expedition) has revealed that on average, viral sequences >5kbp recruited to clustered viral populations with an average of 95% read identity, and reads with an average of <92% identity failed to map. This indicates clear sequence-based delineations between distinct viral populations, and allows for the grouping of viral sequences into ecologically meaningful units [23]. Viral populations are thus defined here as contigs which cluster at 95% identity over 80% of the shorter sequence and which are represented by the longest contig larger than 5kbp.

***Potential auxiliary metabolic genes (AMGs)***

***Assimilatory phosphoadenosine 5’ phosphosulfate (PAPS) reductase***

Structural comparisons of the assimilatory PAPS reductase genes encoded in the two representative contigs revealed similarities with the assimilatory adenosine 5'-2 phosphosulfate reductase (APS) (100% confidence 20%ID and 100% confidence 22%ID, respectively; Supplementary Table 2). APS and PAPS reductases share 25–30% identical amino acids but it is generally accepted that assimilatory PAPS reductase is utilized by bacteria and fungi as opposed to APS that is mainly found in photosynthetic organisms [24].

***AMGs from Mu-like Phages***

***Diguanylate cyclase.*** Signaling pathways are critical for viral induction of host metabolic activities upon infection [25]. Many viruses are known to hijack signaling pathways that control glucose metabolism, protein synthesis, proliferation, and DNA repair [26]. Diguanylate cyclase (DGC) is responsible for the production of the signaling molecule cyclic diguanylate (c-di-GMP), a ubiquitous second messenger in bacteria [27]. We identified a putative DGC on the first of the two Mu-like phage contigs. While the DGC was encoded near the edge of the viral sequence, it was flanked by the phage-like Clp protease on the one side and numerous phage structural genes on the other (Figure 5). The putative DGC gene encodes the PAS_3 conserved domain of DGC and is structurally homologous to a DGC found in Burkholderia spp. (99.8% confidence, 19% ID, Supplementary Table 2) which is also known to be infected by Mu-like phages [28]. A predicted promoter was identified 60 bp upstream of the putative start codon of the DGC gene (Figure 5; Supplementary Table 2). The LDF score of the putative DGC promoter was 0.81indicating that this might be an active promoter of the putative DGC AMG.

***UDP-sulfoquinovose synthase.*** The second Mu-like viral contig (26B_NODE_387), encodes a putative UDP-sulfoquinovose synthase (UDP-SQ) involved in N-glycosylation processes. This gene was located near the center of the sequence and it was bounded on both sides by viral structural and enzymatic genes (Figure 5). The putative UDP-SQ encoded a SQD1_like_SDR_e conserved domain (Supplementary Table 2), with sequence similarity closest to sequences from Deltaproteobacteria (e-value = 1.5e-97). The structural homology of the putative UDP-SQ was similar to tyrosine-dependent oxidoreductases with NAD(P)-binding Rossmann-fold domains (confidence 100%, 43%ID; Supplementary Table 2). UDP-SQ is an enzyme involved in the N-glycosylation process in Crenarchaeota and Euryarchaeota and in sulfolipid biosynthesis in photosynthetic microbes [29,30,31,32]. It produces sulfoquinovose (SQ) which constitutes the headgroup of the sulfolipid sulfoquinovosyl diacylglycerol [33]. Sulfoquinovosyl diacylglycerol is found in different archaeal glycosylated proteins that undergo N-glycosylation (archaella, S-layers, pilin-like proteins) [34,35,36] and has been detected in oxic waters of the Black Sea (~10% of the total intact polar lipids detected) and Eastern Tropical North Pacific (ETNP) (40-50% of the total intact polar lipids detected) ODWC. However, it has not been detected in the suboxic and anoxic interior of ODWCs [37,38].

***Other phage-related viral elements detected in the Cariaco Basin***

***Acetate metabolism.*** Both the Pta and Ack genes identified here are similar to those from *Halomonas* sp. (e-value = 2.5e-158, 4.6e-108). The Pta gene contains the expected Pta conserved domain while the ack gene encodes the requisite acetate kinase A conserved domain (Supplementary Table 2). The Pta protein encoded by our putative Pta AMG is structurally similar to Pta found in *E. coli* (100% confidence, 23%ID) (Supplementary Table 2). The Ack protein encoded by the putative Ack AMG has a similar structural homology with the acetate kinase of Mycobacterium (100% confidence, 23%ID) (Supplementary Table 2). One predicted promoter with LDF score 1.06 was identified ~ 60 bp upstream of the putative prophage pta gene (Supplementary Table 2). The BPROM software also predicted a promoter upstream from the putative Ack ORF with a LDF score of 0.2. However, this prediction should be interpreted with caution, since 0.2 is the minimum score required for a promoter to considered active using BPROM’s LDF score.

***Iron-sulfur cluster formation.*** Iron-sulfur clusters [Fe-S] are important cofactors for a variety of [Fe-S] proteins in bacteria, archaea, and eukaryotes [39]. Due to their chemical properties, they have the remarkable ability to act as modular structures that can facilitate electron transport and influence activity of proteins [40]. The formation of [Fe-S] clusters in cells depends on three distinct systems of bacterial origin: the iron–sulfur cluster (ISC), the sulfur mobilization (SUF) system and NIF system [41].

**References (Supplementary Files)**

1. Noble R, Fuhrman J. Use of SYBR Green I for rapid epifluorescence counts of marine viruses and bacteria. *Aquat Microb Ecol* 1998; **14**: 113-118.

2. Astor YM., Lorenzoni L., Scranton MI. (eds). *Handbook of methods for the analysis of oceanographic parameters at the Cariaco Time Series Station. Cariaco Time Series Study*. Fundación La Salle de Ciencias Naturales: Caracas, Venezuela, 2013.

3. Roux S, Krupovic M, Debroas D, Forterre P, Enaultet F. Assessment of viral community functional potential from viral metagenomes may be hampered by contamination with cellular sequences. *Open Biol.* 2013*;* **3**(12):130160.

4. Hurwitz BL, Deng L, Poulos BT, Sullivan MB. Evaluation of methods to concentrate and purify ocean virus communities through comparative, replicated metagenomics. *Environ Microbiol* 2012; **15**:1428–1440.

5. Enault F, Briet A, Bouteille L, Roux S, Sullivan, MB, Petit MA 2017. Phages rarely encode antibiotic resistance genes: a cautionary tale for virome analyses. *ISME J* **11**: 237-247.

6. Hurwitz BL, Deng L, Poulos BT, Sullivan MB. Evaluation of methods to concentrate and purify ocean virus communities through comparative, replicated metagenomics. *Environ Microbiol* 2013; **15**: 1428–1440.

7. Frias-Lopez J, Shi Y, Tyson GW, Coleman ML, Schuster SC, Chisholm SW et al. Microbial community gene expression in ocean surface waters. *PNAS* 2008; **105**: 3805–3810.

8. Ganesh S, Parris DJ, DeLong EF, Stewart FJ. Metagenomic analysis of size-fractionated picoplankton in a marine oxygen minimum zone. *ISME J* 2014; **8**: 187–211.

9. Suter EA, Pachiadaki M, Taylor GT, Astor Y, Edgcomb VP. Free-living chemoautotrophic and particle-attached heterotrophic prokaryotes dominate microbial assemblages along a pelagic redox gradient. *Environ. Microbiol* 2018; **20**: 693–712.

10. Bolger AM, Lohse M, Usadel B. Trimmomatic: a flexible trimmer for illumina se-quence data. *Bioinformatics* 2014; **30**: 2114–2120.

11. Peng Y, Leung HCM, Yiu SM, Chin FYL. IDBA - A practical iterative De Bruijn graph De Novo assembler. In: Berger B (Ed). *The 14th Annual International Conference on Research in Computational Molecular Biology (RECOMB 2010)*. Springer-Verlag: Berlin, Heidelberg, Germany, 2010 pp 426–440.

12. Hyatt D, LoCascio PF, Hauser LJ, Uberbacher EC. Gene and translation initiation site prediction in metagenomics sequences. *Bioinformatics*, 2012; **28**: 2223–2230.

13. Edgar RC. Search and clustering orders of magnitude faster than BLAST. *Bioinformatics* 2010; **26**: 2460–2461.

14. Kanehisa M, Goto S. KEGG: Kyoto encyclopedia of genes and genomes. *Nucleic Acids Res* 2000; **28**: 27–30.

15. Jones P, Binns D, Chang HY, Fraser M, Li W, McAnulla C, McWilliam H. InterProScan 5: genome-scale protein function classification. *Bioinformatics* 2014; **30**: 1236-40.

16. Quevillon E, Silventoinen V, Pillai S, Harte N, Mulder N, Apweiler R et al. InterProScan: protein domains identifier. *Nucleic Acids Res* 2005; **33**: W116–W120.

17. Daly RA, Borton MA, Wilkins MJ, Hoyt DW, Kountz DJ et al. Microbial metabolisms in a 2.5-km-deep ecosystem created by hydraulic fracturing in shales. *Nat Microbiol* 2016; **1**: 16146.

18. Roux S, Brum JR, Dutilh BE, Sunagawa S, Duhaime MB, Loy A et al. Ecogenomics and potential biogeochemical impacts of globally abundant ocean viruses. *Nature* 2016; **537**: 689–693.

19. Umarov RK, Solovyev VV. Recognition of prokaryotic and eukaryotic promoters using convolutional deep learning neural networks. *PLoS One* 2017; **12**: e0171410.

20. Solovyev V, Salamov A. 2011. Automatic Annotation of Microbial Genomes and Metagenomic Sequences In: Li RW (ed). *Metagenomics and its applications in agriculture* *biomedicine and environmental studies.* Nova Science Publishers, Hauppauge, NY, USA: pp 61–78.

21. Hücker SM, Ardern Z, Goldberg T, Schafferhans A, Bernhofer M, Vestergaard G et al. Discovery of numerous novel small genes in the intergenic regions of the *Escherichia coli* O157:H7 Sakai genome. *PLoS One* 2017; **12**: e0184119.

22. Langmead B, Salzberg SL. Fast gapped-read alignment with Bowtie 2. *Nat Methods* 2012; **9**: 357-359.

23. Gregory AC, Zayed AA, Conceição-Neto N, Temperton B, Bolduc B, Alberti A et al. Marine DNA viral macro- and microdiversity from Pole to Pole. *Cell* 2019; **177**: 1109-1123.

24. Kopriva S, Büchert T, Fritz G, Suter M, Benda R, Schünemann V et al. The presence of an iron-sulfur cluster in adenosine 5'-phosphosulfate reductase separates organisms utilizing adenosine 5'-phosphosulfate and phosphoadenosine 5'-phosphosulfate for sulfate assimilation. J *Biol Chem* 2002; **277**: 21786-91.

25. Sanchez EL, Lagunoff M. Viral activation of cellular metabolism. *Virology* 2015; **479-480**: 609-618.

26. Diehl N, Schaal H. Make yourself at home: viral hijacking of the PI3K/Akt signaling pthway. *Viruses* 2013; **5**: 3192-3212.

27. Jenal U, Malone J. Mechanisms of cyclic-di-GMP signaling in bacteria. *Annu Rev Genet* 2006; **40**: 385-407.

28. Summer EJ, Gonzalez CF, Carlisle T, Mebane LM, Cass AM, Savva CG et al. Burkholderia cenocepacia phage BcepMu and a family of Mu-like phages encoding potential pathogenesis factors. *J Mol Biol* 2004; **340**(1):49-65.

29. Benning C, Somerville CR. Identification of an operon involved in sulfolipid biosynthesis in Rhodobacter sphaeroides. *J Bacteriol* 1992; **174**: 6479–6487.

30. Güler S, Seeliger A, Härtel H, Renger G, Benning C. A null mutant of Synechococcus sp. PCC7942 deficient in the sulfolipid sulfoquinovosyl diacylglycerol. *J Biol Chem* 1996; **271**: 7501–7507.

31. Meyer BH, Zolghadr B, Peyfoon E, Pabst M, Panico M, Morris HR et al. Sulfoquinovose synthase – an important enzyme in the N‐glycosylation pathway of *Sulfolobus acidocaldarius*. *Mol Microb* 2011; **82**: 1150-1163.

32. Zolghadr B, Gasselhuber B, Windwarder M, Pabst M, Kracher D, Kerndl M et al (2015). UDP-sulfoquinovose formation by Sulfolobus acidocaldarius. *Extremophiles* 2015; **19**: 451-67.

33. Benning C. Biosynthesis and function of the sulfolipid sulfoquinovosyl diacyglycerol. *Annu Rev Plant Physiol Plant Mol Biol* 1998; **49**: 53-75.

34. Ng SY, Wu J, Nair DB, Logan SM, Robotham A, Tessier L et al. Genetic and mass spectrometry analyses of the unusual type IV-like pili of the archaeon Methanococcus maripaludis. *J Bacteriol* 2011; **193**:804–814.

35. Tripepi M, You J, Temel S, Onder O, Brisson D, Pohlschröder M. N-glycosylation of *Haloferax volcanii* flagellins requires known Agl proteins and is essential for biosynthesis of stable flagella. *J Bacteriol* 2012; **194**: 4876–4887.

36. Palmieri G, Balestrieri M, Peter-Katalinić J, Pohlentz G, Rossi M, Fiume I. Surface-exposed glycoproteins of hyperthermophilic Sulfolobus solfataricus P2 show a common N-glycosylation profile. *J Proteome Res* 2013; **12**: 2779-90.

37. Schubotz F, Wakeham SG, Lipp JS, Fredricks HF, Hinrichs KU. Detection of microbial biomass by intact polar membrane lipid analysis in the water column and surface sediments of the Black Sea. *Environ Microb* 2009; **11**(10): 2720-2734.

38. Schubotz F, Xie S, Lipp JS, Hinrichs KW, Wakeham SG. Intact polar lipids in the water column of the eastern tropical North Pacific: abundance and structural variety of non-phosphorus lipids. *Biogeosciences* 2018; **15**: 6481–6501.

39. Meyer J. Iron-sulfur protein folds, iron-sulfur chemistry, and evolution. *J Biol Inorg Chem* 2008; **13**: 157-70.

40. Beinert H, Holm RH, Münck E. Iron-sulfur clusters: nature's modular, multipurpose structures. *Science* 1997; **277**: 653-9.

41. Lill R. Function and biogenesis iron–sulphur proteins. *Nature* 2009; **460**: 831–838.
