# Supplementary figures and images for: Viral elements and their potential influence on microbial processes along the permanently stratified Cariaco Basin redoxcline
Source: ISME J. 2020 Aug 14;14(12):3079–92. doi: 10.1038/s41396-020-00739-3 (PMC7785012; doi:10.1038/s41396-020-00739-3)

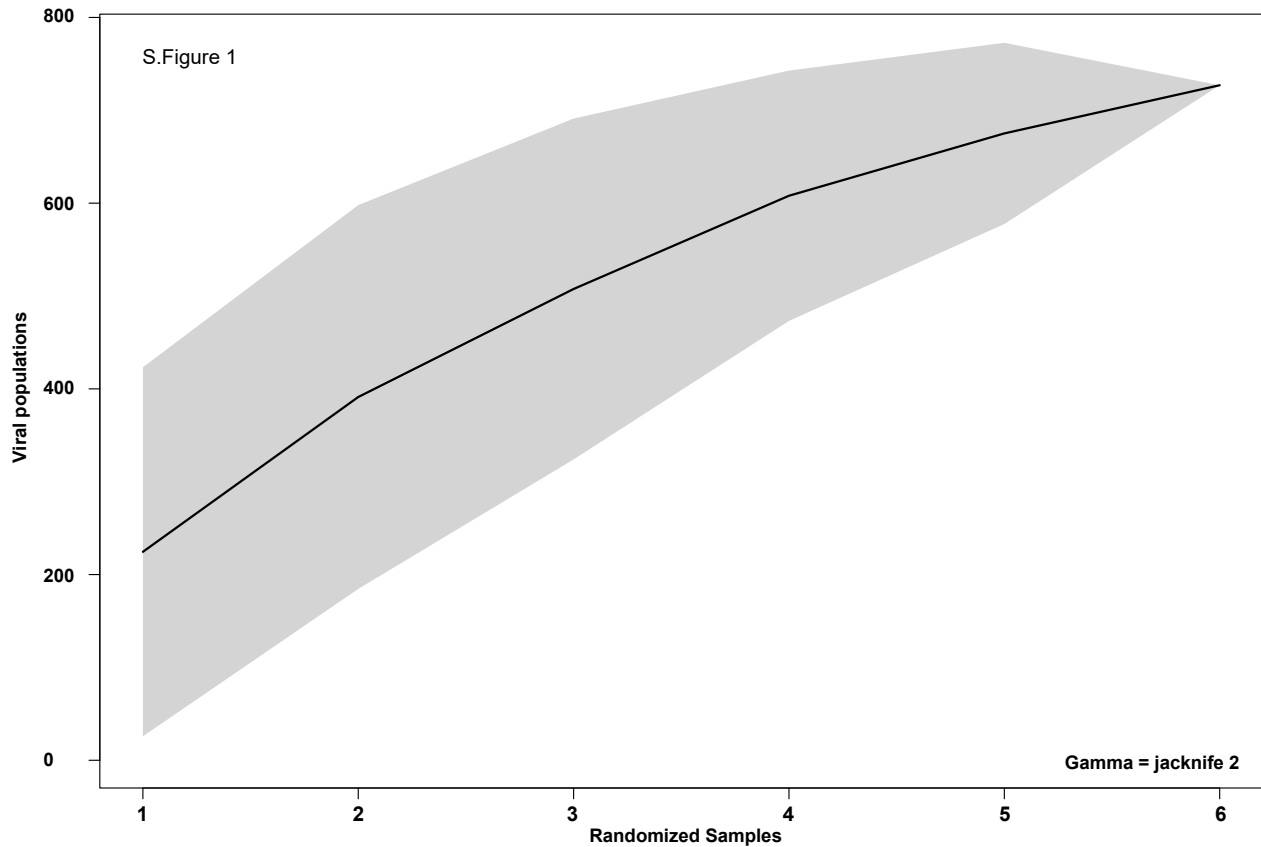

Supplement: Supplementary file 2 — Supplementary Figure 1 [file 41396_2020_739_MOESM2_ESM.pdf]

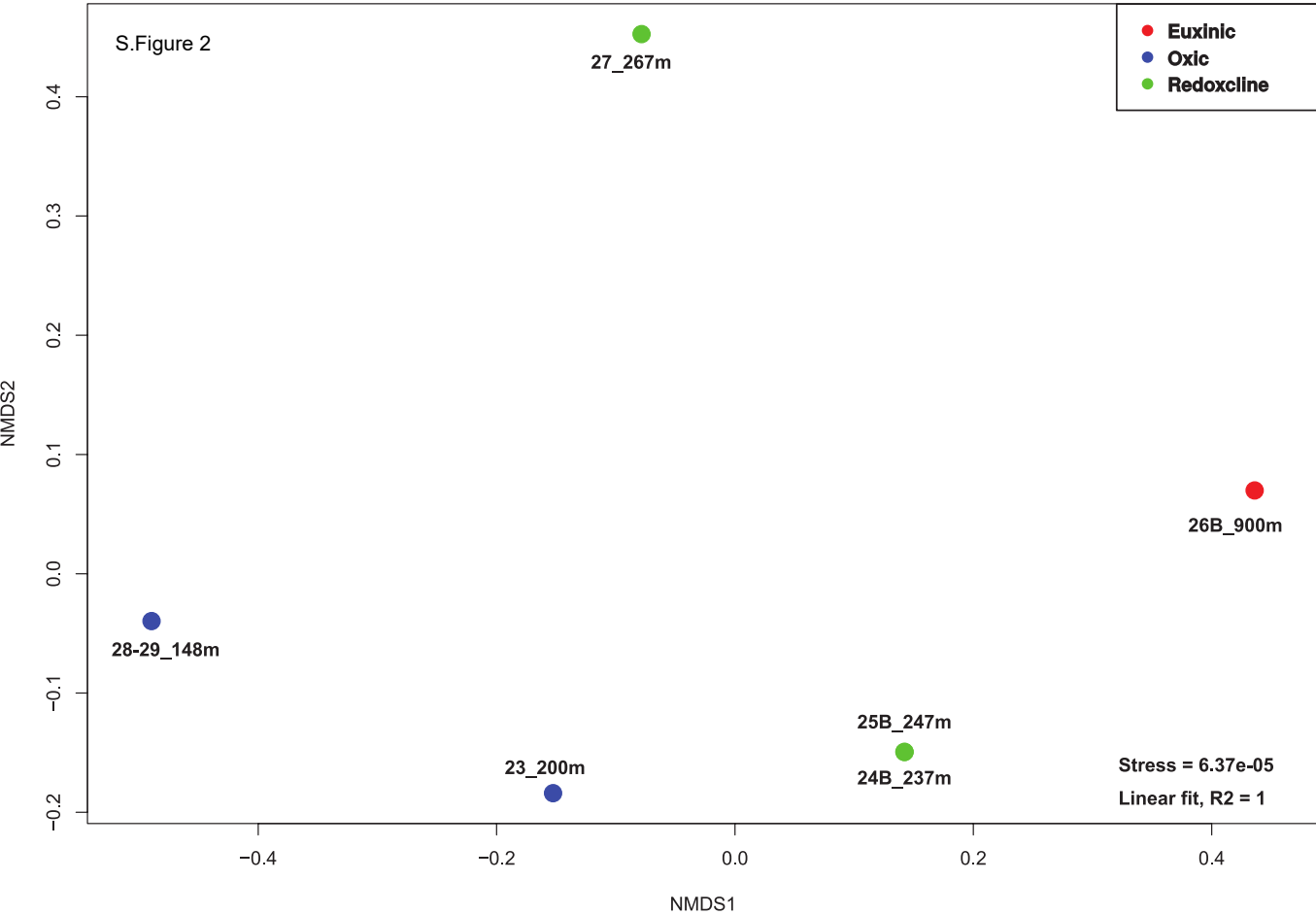

Supplement: Supplementary file 3 — Supplementary Figure 2 [file 41396_2020_739_MOESM3_ESM.pdf]

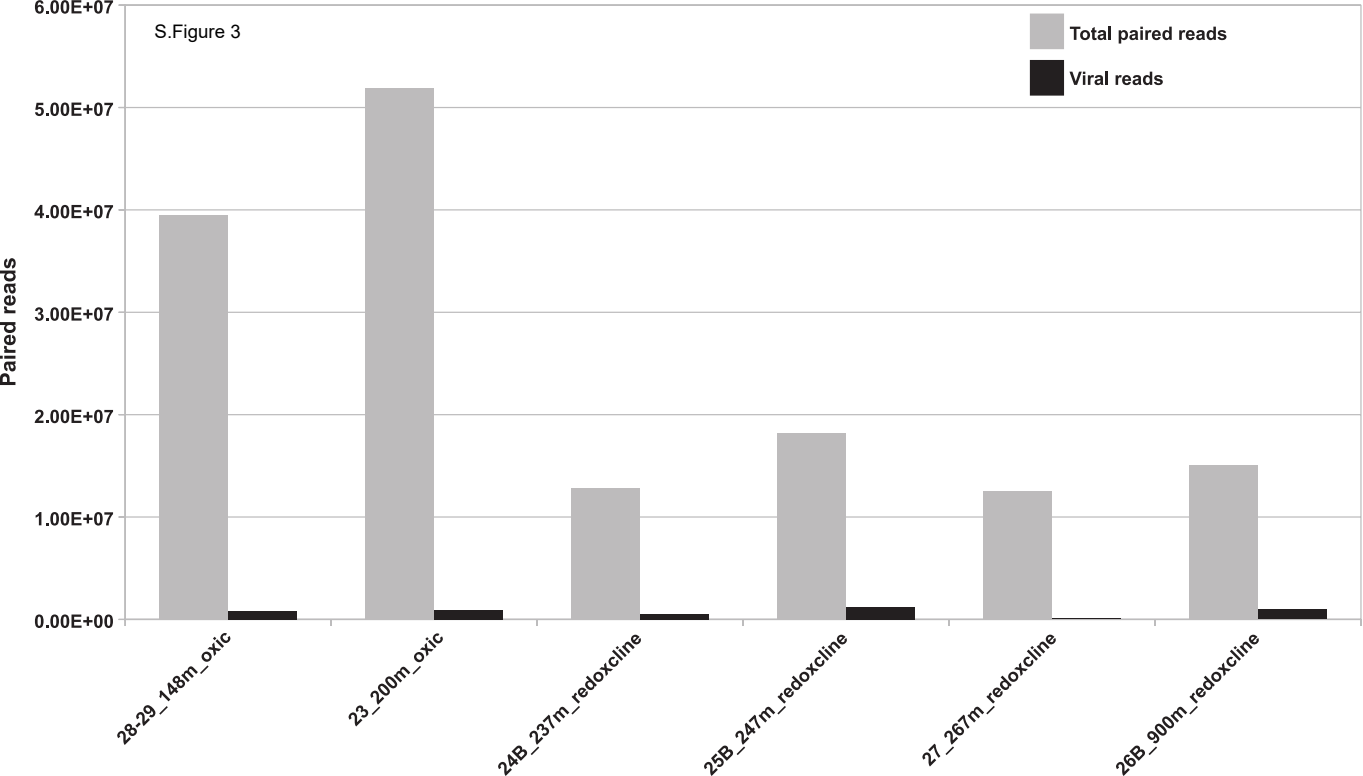

Supplement: Supplementary file 4 — Supplementary Figure 3 [file 41396_2020_739_MOESM4_ESM.pdf]

S.Figure 4

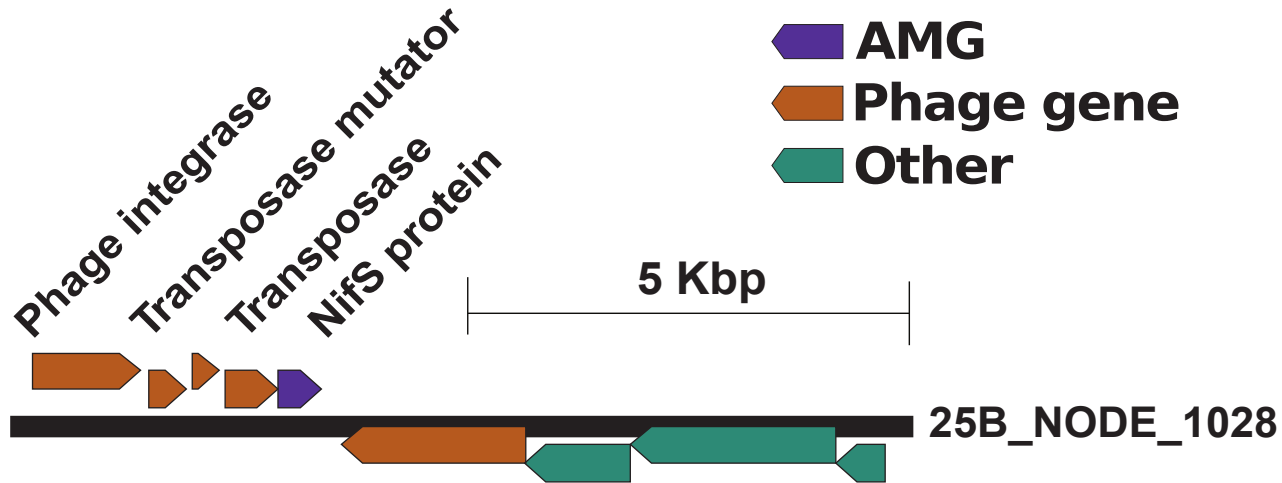

Supplement: Supplementary file 5 — Supplementary Figure 4 [file 41396_2020_739_MOESM5_ESM.pdf]
