# Supplementary material for: Viral elements and their potential influence on microbial processes along the permanently stratified Cariaco Basin redoxcline
Source: ISME J. 2020 Aug 14;14(12):3079–92. doi: 10.1038/s41396-020-00739-3 (PMC7785012; doi:10.1038/s41396-020-00739-3)
Supplement: Supplementary file 6 — Supplementary Table 1 [file 41396_2020_739_MOESM6_ESM.pdf]

S. Table 1

Depth (m)	Oxygen (µM) CTD	Redox	mean prokaryote abundance (10^8/L)	mean virus-like particles (VLP) counts (10^8/L)	volume concentrated for the virome (L)	sulfide** (µM)	NO_3** (µM)	NO_2** (µM)	NH_4** (µM)
148	81.4	oxic	0.787	5.85	10	0	12	0.07	2
200	13.3	oxic	0.898	3.03	18	0	11.2	0.08	1.4
237*	0.36	redoxcline	0.903	4.74	16	0	3	0.23	0.6
247*	undetectable	redoxcline	1.556	4.44	18	0	2.5	0.13	0.2
267	undetectable	redoxcline	0.736	4.19	18	0.4	0.1	0.03	0.5
900*	undetectable	euxinic	0.469	3.23	16	87	0	0	26.9

*samples treated with CsCl

**data collected 3 days after the viral samples
