# Supplementary material for: Viral elements and their potential influence on microbial processes along the permanently stratified Cariaco Basin redoxcline
Source: ISME J. 2020 Aug 14;14(12):3079–92. doi: 10.1038/s41396-020-00739-3 (PMC7785012; doi:10.1038/s41396-020-00739-3)
Supplement: Supplementary file 9 — Supplementary Table 4 [file 41396_2020_739_MOESM9_ESM.pdf]

S. Table 4

Depth (m)	Oxygen condition	Sequencing depth (reads)	Viral reads recruited	Total raw contigs	Contigs >1.5kb clustered 95%ID 80% sequence	Identified as viral	Populations > then 5kb (observed species richness)	Evenness	Inverse Simpson’s concentration
148	oxic	39521714	853049	123907	3023	510	71	0.99	123.65
200	oxic	51912321	918640	189211	6771	331	75	0.99	241.49
237	redoxcline	12823928	556452	156872	4109	567	174	0.99	324.81
247	redoxcline	18228362	1176756	155307	4448	204	72	0.99	209.58
267	redoxcline	12513936	89872	57487	2202	33	13	0.99	53.18
900	euxinic	15084372	968170	300231	7560	587	242	0.99	327.68
